# Hydronaphthol—A New Antiseptic

**Published:** 1886-02

**Authors:** 


### Hydronaphthol—A New Antiseptic.

In an article on the antiseptic properties of this new substance, Dr. George R. Fowler, of New York, gives the following history:

Hydronaphthol belongs to the phenol series, and bears the same relation to naphthyl, the hypothetical compound radical of naphthalin that carbolic acid does to the compound radical phenyl, Thus, carbolic acid was formerly regarded as the hydrated oxide of phenyl. Hydronaphthol, considered in the same way, would be a hydrated oxide of naphthyl. At the present time, however. these hypothetical compounds, phenyl and napththyl, are considered as obsolete, and not capable of existing. In fact, carbolic acid is regarded as an oxide of benzol, or as a benzol in which one equivalent of the hydrogen is substituted by one of hydroxyl

(O H). Naphthol is obtained from the sodium naphtholate by decomposing it with hydrochloric or sulphuric acid; it is then purified by distillation.

Hydronaphthol is a derivative of the hydroxyl substitute of naphthalin, which latter of itself possesses antiseptic properties of sufficient value to have already excited notice and a desire to learn more of its compounds. The term "hydronaphthol," though perhaps not, strictly speaking, correct, yet conveys sufficiently well its character and relations to naphthalin, and at the same time is a convenient term for every-day use. It has been but recently discovered that it possesses antiseptic properties, and the claim is made that it is from ten to fifteen times more efficacious than carbolic acid. It is the most prominent antiseptic of the phenol series, and, besides, possesses so many other advantages over substances now used for this purpose that it bids fair to supersede many of these. In surgical practice it will take the place, probably, of carbolic acid. Of the many new members of the phenol series which have been discovered since Calvert called attention to carbolic acid about thirty years ago, and which have been utilized in the industrial arts, some are better antiseptics than the latter. With but one or two exceptions, however, none have obtained any prominence as germicidal agents. Carbolic acid, though a fairly reliable antiseptic in strong solutions, when so used, involves some risk to life, from its corrosive action upon animal tissues and well-known poisonous properties. In weak solutions it is exceedingly unreliable, and its disagreable odor often hides that of putrefaction, instead of preventing the occurrence of the latter. On the other hand, hydronaphthol is non-irritant, non-poisonous, and non-corrosive; and, although only soluble in water to the extent of one part in one thousand, in this proportion is antiseptic. It has no odor to disguise that of putrefaction, nor is it decomposed nor rendered inert by the products of putrefactive decomposition—such as sulphureted hydrogen, ammonia, etc. It is far more stable than carbolic acid, not being volatile at ordinary temperature. Its vapor, when volatilized for purposes of fumigation, has no obnoxious effect upon the organs of respiration. It will not injure, either in substance, solution, or vapor, colors or textile fabrics. Its sparing solubility in water is rather an advantage than otherwise, as mistakes in making solutions can not occur. A saturated solution is about of the strength of one to one thousand, and in this proportion it will perfectly preserve for an indefinite time animal tissues and fluids, and yet upon living tissues this solution produces no perceptible effect other than the formation of a very slight albuminate film—this latter to be considered rather an advantage than otherwise, inasmuch as it constitutes an additional security against infectious germs floating in the air. If for no other reason than that it is non-corrosive, and hence will not injure the polished surface and keen edge of cutting instruments, it is to be preferred to mercuric bi-chloride, and to the latter it is second only in antiseptic qualities. It has a slight aromatic taste and odor, and crystallizes in scale like clinorhomboid laminæ of a silvery white or grayish hue. Although but sparingly soluble in water, it dissolves freely in alcohol, ether, chloroform, glycerine, benzole, and the fixed oils. It is not volatile at ordinary temperatures, but begins to sublime at about 90° C. With the alkalies and the alkaline earths it forms compounds which are unstable, are readily decomposed by carbonic acid, and of doubtful antiseptic value. It is easily powdered, and in this condition, triturated with carbonate of magnesia, silicates, such as fuller's earth, China clay, etc., in the proportion of two parts of the hydronaphthol to one hundred of either of the above named, can be dusted along the line of incision and over the mouths of drainage tubes, in the latter application having an advantage over iodoform, now so commonly used for that purpose, in that it does not dry up the serum escaping from the wound cavity, and thus block up the exit extremity of the tube. Absorbent gauze, cotton, jute, wood-flour, sawdust, peat, moss, and paper-wool may be impregnated with it by immersing them in its alcoholic or benzole solution and then drying; the hydronaphthol crystals cling to these without the aid of stearin, paraffin, or resin, as in the case of carbolic acid. As it is not decomposed by the presence of organic matter, it possesses this advantage over corrosive sublimate in the preparation of surgical dressings. Its ten-per-cent alcholic solution perfectly sterilizes silk, and sufficiently hardens and preserves, as well as sterilizes, catgut.—*New York Medical Journal.*

### Periods of Growth—A New Start in Physiological Research.

During the International Medical Conference, held in Copenhagen in the summer of 1884, a paper read by the Rev. Malling Hansen, Principal of the Danish Institution for the Deaf and Dumb, was listened to with marked attention and interest. It gave the results of the daily weighings and measurements of height which he had carried on for nearly three years on the one hundred and thirty pupils—seventy-two boys and fifty-eight girls—of the institution, and demonstrated facts as to the development of the human body during the period of childhood that perfectly startled and astonished the assembled medical authorities, opening an entirely new field for investigation and reflection. Since then Mr. Hansen has continued his observations, and though he has yet a tremendous amount of work before him, he believes himself able to state now the outlines of the results he has obtained. Before entering upon these it may be proper to explain that the children are weighed four times daily, in batches of twenty—in the morning, before dinner, after dinner and at bedtime, and that each child is measured once a day. The scales and appliances used are ingeniously contrived, allowing the whole business to be finished in five to ten minutes, and sufficiently accurate to indicate even the smallest difference. The children have taken immensely to the thing; they are eager to perform their part, and all fingers are astir to repeat the numbers, rejoicing at every marked change.

The common impression is, no doubt, that increase in bulk and height of the human body during the year of growth progresses evenly all through the year. This is not so. Three distinct periods are marked out, and within them some thirty lesser waverings have been observed. As for bulk, the *maximum* period extends from August until December; the period of equipoise lasts from December until about the middle of April; and then follows the *minimum* period until August. The lasting increase of bulk or weight is all accumulated during the first stage; the period of equipoise adds to the body about a fourth of that increase, but this gain is almost entirely spent or lost again in the last period. The increase in the height of children shows the same division into periods, only in a different order. The *maximum* period of growth in height corresponds to the *minimum* period of increase in bulk, and vice versa. In September and October a child grows only a fifth of what it did in June and July. In other words, during a part of the year, autumn and beginning of winter, the child accumulates bulk, but the height is stationary; in the early summer the bulk remains nearly unchanged, but the vital force and the nourishment are expended to the benefit of height. While the body works for bulk there is rest for the growth, and when the period of growth comes the working for bulk is suspended.

The human body has, consequently, the same distinctly-marked periods of development as the plants. Mr. Hansen has extended his daily measurements also to a number of trees in the garden of the institution, and has convinced himself that here also a period of growth in length, as represented by the branches, twigs and tops, alternates with another of increase in bulk—that is, in the circumference of the trunk, followed by a third period of equipoise or rest. In April and May the entire force of the tree was expended in lengthening the branches, while the thickness of the trunk remained stationary; all through May the most exact measurement failed to discover any increase of bulk. But in June until the middle of July, when the new twigs had been all formed, it was the trunk that absorbed the nourishment from the roots and bulged out. Then came the period of rest and inactivity.

It was stated above that, within the three chief periods of the human (or rather the infantile) body, the observations had pointed out some thirty smaller waverings of a more passing nature. The ordinary movement may be subjected to disturbances, a swifter or a slower increase, sometimes even positive loss. These waverings prove to be subject to constant laws and independent of accidental or local influences; they return exactly in the same manner and present the same features. One constant relation can be pointed out between these waverings and the movements of temperature in the outer air. If the thermometer rises steadily through several days, the increase in weight also become greater, but not equally from day to day; the second day the increase becomes double, the third day three times as much, and so on, until there is a fall of the temperature. If it grows colder there is a decrease in weight (or a lesser increase), but again on the second day double, the third day three times as much, and so on, until a new change of temperature intervenes. The effect of the short periods of change in the thermometer is, consequently, just the contrary of that observed in the longer periods. A higher temperature is favorable to increase in weight or bulk; a lower counteracts it, if considered singly; while the warmer seasons, spring and summer, put a stop to that form of growth, leaving it to autumn and winter to favor it.

Mr. Hansen has not restricted himself to the set of pupils in the institution intrusted to this care. He has caused corresponding observations to be made also in several other places where a number of children were subjected to the same regime and the same conditions of life. An orphan school in Copenhagen, another deaf mute institution in Jutland, and a village school have under his instructions carried out a series of investigations, for which a subvention has been granted out of the public funds. The results obtained confirm in a most surprising manner his own. In the graphic representation of the observations the curves from the different fields of investigation follow each other very closely, mounting and descending under the evident influence of identical laws. He is still engaged upon his arduous task and working out the many different problems meeting him on his way. Physiologists will, however, hardly be inclined to accept all his facts or to subscribe to all his deductions. It is only right that they judge for themselves, verify the observations, and submit the deductions from them to a careful trial. If it turns out that they are proved, then various conclusions of high importance for the hygienic treatment of children are to be drawn. At all events, Mr. Hansen may claim to have opened an entirely new and undoubtedly fertile field for exact physiological research.—*London Times.*

